# Plastic deformation behaviour of single-crystalline martensite of Ti-Nb shape memory alloy

**DOI:** 10.1038/s41598-017-15877-6

**Published:** 2017-11-16

**Authors:** Masaki Tahara, Nao Okano, Tomonari Inamura, Hideki Hosoda

**Affiliations:** 1 Laboratory for Materials and Structures, Institute of Innovative Research, Tokyo Institute of Technology, 4259 Nagatsutacho, Midori-ku, Yokohama, 226-8503 Japan; 2 Laboratory for Future Interdisciplinary Research of Science and Technology, Institute of Innovative Research, Tokyo Institute of Technology, 4259 Nagatsutacho, Midori-ku, Yokohama, 226-8503 Japan; 3 Graduate Student, Tokyo Institute of Technology, 4259 Nagatsutacho, Midori-ku, Yokohama, 226-8503 Japan; 4 Present Address: Nippon Steel & Sumitomo Metal Corporation, 1-8 Fusoucho, Amagasaki, Hyogo, 660-0891 Japan

## Abstract

β-Ti alloys have attracted considerable attention as new biomedical shape memory alloys. Given the critical importance of the plastic deformation in the martensite phase for the shape memory effect and superelasticity, we investigated here the plastic deformation behaviour of a single crystal of α″ (orthorhombic) martensite of Ti-27 mol%Nb shape memory alloy obtained by the stress-induced martensitic transformation of a single crystal of the parent β phase. Four operative plastic deformation modes were observed, including two dislocation slips and two twinnings. To the best of our knowledge, two of these plastic deformation modes (one dislocation slip and one twinning) were discovered for the first time in this study. The identified slip and twinning systems in the martensite phase have corresponding slip and twinning systems in the parent β phase with which they share many similarities. Therefore, we believe that the plastic deformation of the α″ martensite is inherited from that of the parent β phase.

## Introduction

Noble Ti alloys have been actively investigated in the past two decades owing to their high biocompatibility and unique mechanical properties, which make them a choice material for biomedical applications^1–4^. In particular, Ti base shape memory alloys (SMAs) composed of non-toxic elements have attracted considerable attention as new biomedical SMAs. The most widely applied SMA in the biomedical field is Ni-Ti alloy. However, the risk of triggering an allergic reaction to Ni by using a Ni-Ti SMA has been pointed out^5,6^. To address this issue, Ni-free Ti base SMAs have been developed in recent years. The shape memory effect and superelasticity in Ti base SMAs are due to the martensitic transformation between the β phase (bcc, parent phase) and the α″ phase (orthorhombic, martensite phase). The research on the material design (e.g. optimisation of alloying elements^7–16^ and thermo-mechanical treatments^17–19^) and fundamental aspects of martensitic transformation^20–29^ in Ti base alloys has rapidly grown. One of the biggest problem of Ti base SMAs from a practical viewpoint is a low critical stress for plastic deformation, which causes a small shape recovery strain^9^ and unstable superelasticity during the cyclic deformation^30–32^. While a lot of effort has been dedicated to increasing the critical stress for plastic deformation^8–10,14,18^, there have been only a few reports on the plastic deformation modes in Ti base SMAs. The plastic deformation of metastable β-Ti alloys in the β phase has been systematically investigated by many researchers^33–38^, in particular the effect of the loading orientation, test temperature and alloy composition on the main plastic deformation modes (i.e. dislocation slip with a Burgers vector ***b*** parallel to <111>_b_ and ${\{332\}}_{{\rm{b}}}{\langle 11\bar{3}\rangle }_{{\rm{b}}}$ twin, with the subscript ‘b’ denoting the parent β phase (bcc)) in single-crystalline samples. However, the plastic deformation in the martensite phase is critically important for the shape memory effect and superelasticity because the plastic deformation of SMAs normally occurs during the deformation of reoriented or stress-induced martensite variants^39,40^. Previous limited reports have suggested that the dislocation slip with ***b***//[110]_o_^41^ and ${\{130\}}_{{\rm{o}}}{\langle \bar{3}10\rangle }_{{\rm{o}}}$ twin^41–44^, with the subscript ‘o’ denoting the α″ martensitic phase (orthorhombic), are operative in the α″ martensite phase of Ti base alloys.

One of the reasons for the limited number of reports on the plastic deformation of α″ martensite is the difficulty in making single-crystalline martensite samples, which are nonetheless very helpful for analysing plastic deformation modes. Even when a single crystal of the parent β phase is cooled below the martensitic transformation temperature, an α″ martensite crystal with multi-variants forms. This type of structure is called a self-accommodated microstructure, i.e. without any apparent change in shape. Here, we focused on the stress-induced martensitic transformation that enables to selectively grow a specific variant of martensite from the parent phase. This variant selection by external stress is at the origin of the transformation strain and superelasticity in shape memory alloys^39^. Otsuka and co-workers^45–48^ have succeeded in making and investigating the deformation behaviour of single-crystalline martensite of Cu-Al-Ni SMAs by this method.

In this study, the binary alloy Ti-27 mol%Nb was chosen because it is known to undergo a stress-induced martensitic transformation at room temperature^9,44,49^. Single-crystalline α″ martensite of Ti-27 mol%Nb was obtained here by a stress-induced martensitic transformation from a single crystal of the parent β phase, and its plastic deformation behaviour in terms of crystallographic orientation dependence and deformation modes was systematically investigated.

## Results

### Formation of single crystalline α″ martensite by compression

The single-crystalline samples at room temperature before the compression tests were of the parent β phase (see Supplementary Fig. S1), in agreement with previous reports^9,44,49^. The chemical composition analyses (see Supplementary Table S1) showed a Nb content of almost 27 mol% and a low content of impurities such as oxygen and nitrogen. Given this chemical composition, the samples were expected to exhibit a stress-induced martensitic transformation. Eleven different compression axes were chosen in this study, corresponding to 11 different samples hereafter referred to as Samples #1–#11, and these are listed in Table 1 using the coordinates of the parent β phase.

**Table 1:** Compression axis, observed habit planes, stress-induced CV(s), second yielding stress (*σ*_slip_) and Schmid factor. In Samples #1–#6, single crystals of α″ martensite with CV5 were obtained.

Sample Number	Compression axis in β phase	Favorable CV predicted by *U*	Observed habit plane	Stress-induced CV(s)	Compression axis in α″ phase	*σ*_slip_ (MPa)	Schmid factor	
							[110]_o_	[101]_o_
#1	$[\bar{1}28]$_b_	CV5	$(\overline{0.45},\overline{0.57},0.68)$_b_	CV5	$[0.98,0.06,0.18]$_o_	267	0.486	0.496*
#2	$[\bar{1}25]$_b_	CV5	$(\overline{0.50},\overline{0.52},0.70)$_b_	CV5	$[0.95,0.09,0.28]$_o_	315	0.487*	0.459
#3	$[\bar{1}48]$_b_	CV5	$(0.47,0.51,0.72)$_b_	CV5	$[0.94,0.17,0.29]$_o_	343	0.499*	0.463*
#4	$[\bar{1}23]$_b_	CV5	$(0.55,0.51,0.66)$_b_	CV5	$[0.89,0.14,0.44]$_o_	375	0.481*	0.356
#5	$[\bar{1}34]$_b_	CV5	$(0.46,0.45,0.77)$_b_	CV5	$[0.88,0.21,0.43]$_o_	335	0.498	0.389*
#6	$[\bar{7}811]$_b_	CV5	$(\overline{0.49},\overline{0.52},0.70)$_b_	CV5	$[0.82,0.04,0.56]$_o_	455	0.398*	0.189
#7	$[001]$_b_	CV5, CV6	$(\overline{0.52},\overline{0.49},0.70)$_b_	CV5	—	—	—	—
			$(\overline{0.54},0.50,0.68)$_b_	CV6				
#8	$[013]$_b_	CV5, CV6	$(0.49,0.52,0.70)$_b_	CV5	—	—	—	—
			$(\bar{0.53},\,0.48,\,0.70)$_b_	CV6				
#9	$[035]$_b_	CV5, CV6	$(0.54,0.47,0.70)$_b_	CV5	—	—	—	—
			$(\overline{0.49},0.48,0.73)$_b_	CV6				
#10	$[\bar{1}\,2\,11]$_b_	CV5	$(0.51,0.50,0.70)$_b_	CV5	—	—	—	—
			$(\overline{0.46},0.47,0.75)$_b_	CV6				
#11	$[\bar{1}11]$_b_	CV2, CV3, CV5	$(0.69,0.49,\overline{0.53})$_b_	CV2	—	—	—	—
			$(\overline{0.59},0.65,\overline{0.47})$_b_	CV3				
			$(\overline{0.50},\overline{0.48},0.73)$_b_	CV5				

The compression axis in the α″ martensite phase, *σ*_slip_ and Schmid factor for the MRSSP are indicated for these six samples. Operated slip systems are indicated by an asterisk (*) in the column of Schmid factor.

The lattice correspondence between the α″ martensite and the parent β phases in β-Ti alloys is similar to that in Au-Cd alloys^50^, and can be expressed as follows:

1 $${[100]}_{{\rm{o}}}// < 100{ > }_{{\rm{b}}};{[010]}_{{\rm{o}}}// < 011{ > }_{{\rm{b}}};\,{[001]}_{{\rm{o}}}// < 0\bar{1}1{ > }_{{\rm{b}}}$$

According to this lattice correspondence, there are six lattice corresponding variants (CVs) (see Supplementary Table S2). These six CVs are equivalently formed in thermally induced martensitic transformation, leading to self-accommodation. On the other hand, in the case of stress-induced martensitic transformation, specific CVs of martensite are selectively formed owing to the uniaxial external stress. Favourable CVs induced by the external stress reduce the potential energy of the applied stress. They can therefore be predicted by calculating the interaction energy (*U*) between the uniaxial compression stress and the lattice deformation strain along the compression axis. The CV(s) with the largest negative value of *U* is (are) then the favourable variant(s)^51–54^. The favourable CV(s) expected to be formed in this study were determined from the calculated values of *U* for each compression axis (see Supplementary Fig. S2), and are listed in Table 1. The formation of the single variant corresponding to a martensite single crystal was expected in Samples #1–#6 and #10.

**Figure 1:**
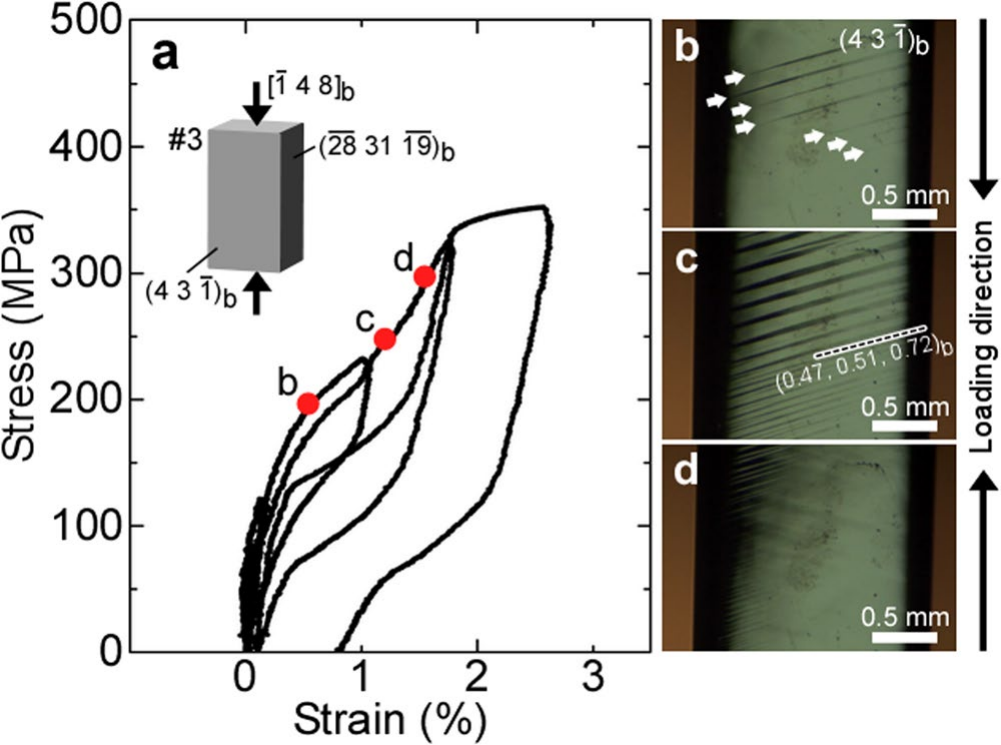
Formation process of single-crystalline α″ martensite by the stress-induced martensitic transformation. (**a**) Stress-strain curves obtained by cyclic loading-unloading compression test for Sample #3 (compression axis = ${[\bar{1}48]}_{{\rm{b}}}$). (**b**), (**c**) and (**d**) are *in situ* OM micrographs of the ${(43\bar{1})}_{{\rm{b}}}$ surface and correspond to the points b, c, and d in (**a**), respectively. Plate-shaped stress-induced α″ martensite crystals with the habit plane (0.47, 0.51, 0.72)_b_ were observed, and the entire sample eventually became a single crystal of α″ martensite (i.e. with a single variant) by compression.

The cyclic loading-unloading compression tests were performed at room temperature, and the stress-induced martensitic transformation was observed in all the samples. The stress-strain curves of Sample #3 are shown in Fig. 1a, and those of the other samples are shown in Supplementary Fig. S3. Two adjacent surfaces parallel to the compression axis were observed by *in-situ* optical microscopy (OM), e.g. the ${(43\bar{1})}_{{\rm{b}}}$ and ${(\bar{28}31\bar{19})}_{{\rm{b}}}$ surfaces were observed in Sample #3. Figure 1b,c and d show the corresponding micrographs of the ${(43\bar{1})}_{{\rm{b}}}$ surface observed during loading at the points b, c and d indicated in the stress-strain curves (Fig. 1a), respectively. Surface reliefs associated with the stress-induced martensitic transformation were formed on the sample surface, as shown in Fig. 1b where the white arrows indicate plate-shaped martensite crystals formed after the first yielding. By further increasing the stress, the stress-induced martensitic transformation progressed, until the entire sample eventually was of the α″ martensite phase (Fig. 1c and d). In many SMAs, the plate-shaped martensite (=habit plane variant) consists of two CVs (major and minor CVs), and these CVs are connected by twinning, thus the minor CV in habit plane variant is called as an internal twin. The internal twins have been frequently observed in the habit plane variant of the self-accommodated martensite of β-Ti SMAs^20^, while in none of the martensite plates could we observe internal twins in this study, indicating that the stress-induced martensite plate consisted of a single CV. This means that the stress-induced martensite plate in this study was single crystalline. In all the samples, the habit plane(s) of the plate-shaped martensite was determined by a two-face trace analysis. The results are shown in Table 1. In addition, the two habit planes corresponding to each CV were calculated by using the phenomenological theory of martensite crystallography (PTMC)^55–57^. As is shown in Supplementary Table S2, the calculation yielded a family of habit planes of the α″ martensite phase approaching the {557}_b_ family, in good agreement with the experimental results shown in Table 1. In the case of Sample #3, only one type of martensite plates, namely that with a (0.47, 0.51, 0.72)_b_ habit plane, was observed, which corresponds to CV5 (see Supplementary Table S2). For all the samples, the stress-induced CV(s) of the martensitic phase corresponding to the observed habit planes is(are) shown in the fifth column of Table 1. The predictions by calculating *U* matched the experimental observations for Samples #1–#9 and #11. On the other hand, Sample #10 was expected to become only CV5, but both CV5 and CV6 were obtained. The values of *U* for CV5 and CV6 are very close, as is shown in Supplementary Fig. S2j, therefore both of these CVs were induced by compression stress in Sample #10. Samples #1–#6 consisted of a single variant of α″ martensite after stress-induced martensitic transformation, which means that they were all single crystalline.

**Figure 2:**
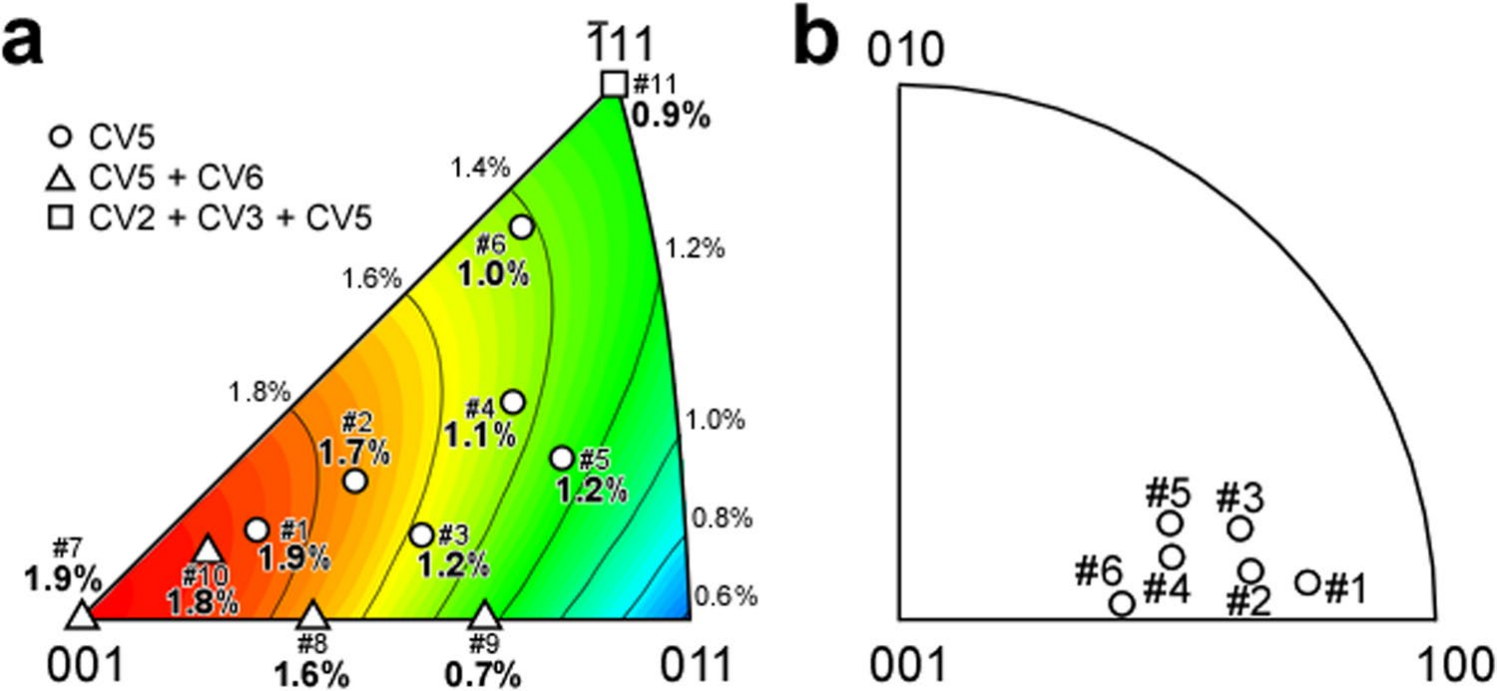
Compression axis in the parent β phase (before compression) and α″ martensite phase. (**a**) Compression axis of eleven samples in the parent β phase in the standard stereographic triangle. The relationship between the maximum lattice deformation strain by compression (contour lines) and the obtained transformation strain due to the martensitic transformation is indicated. (**b**) Compression axis of Samples #1–#6 in the α″ martensite phase.

The relationship between the compression axis in the parent β phase and stress-induced CV(s) is shown in a standard stereographic triangle of the parent β phase in Fig. 2a. The contour lines indicate the value of lattice-deformation strain along the compression axis due to the compression-stress-induced martensitic transformation. These values were calculated using the lattice parameters and orientation relationship between the β and α″ phases. The experimental values of the transformation strain (indicated by a white circle, triangle or square in Fig. 2a) were evaluated from the stress-strain curves for each alloy (see Supplementary Fig. S3). As can be seen in Fig. 2a, they display good agreement with the calculated values. Single-crystalline α″ martensite was obtained when the compression axis was in the central region of the standard stereographic triangle. The compression axis was calculated for Samples #1–#6 in the lattice coordinates of α″ martensite, and is given in Table 1 and Fig. 2b.

It has been reported that Ti-27 mol%Nb polycrystals exhibit superelasticity at room temperature^9,44,49^. However, in Samples #1, #6 and #8, the stress-induced α″ martensite could not reversely transform into the parent β phase by unloading (see Supplementary Fig. S3). This is due to a slight decrease in the number of impurities and Nb content in these three samples (see Supplementary Table S1), which resulted in an increased martensitic transformation temperature^8–10,12–14^. Nevertheless, this slight change in the martensitic transformation temperature did not affect the analysis of the plastic deformation modes of stress-induced single-crystalline α″ martensite with CV5 (Samples #1–#6) investigated in the next section.

### Plastic deformation of single-crystalline α″ martensite

**Figure 3:**
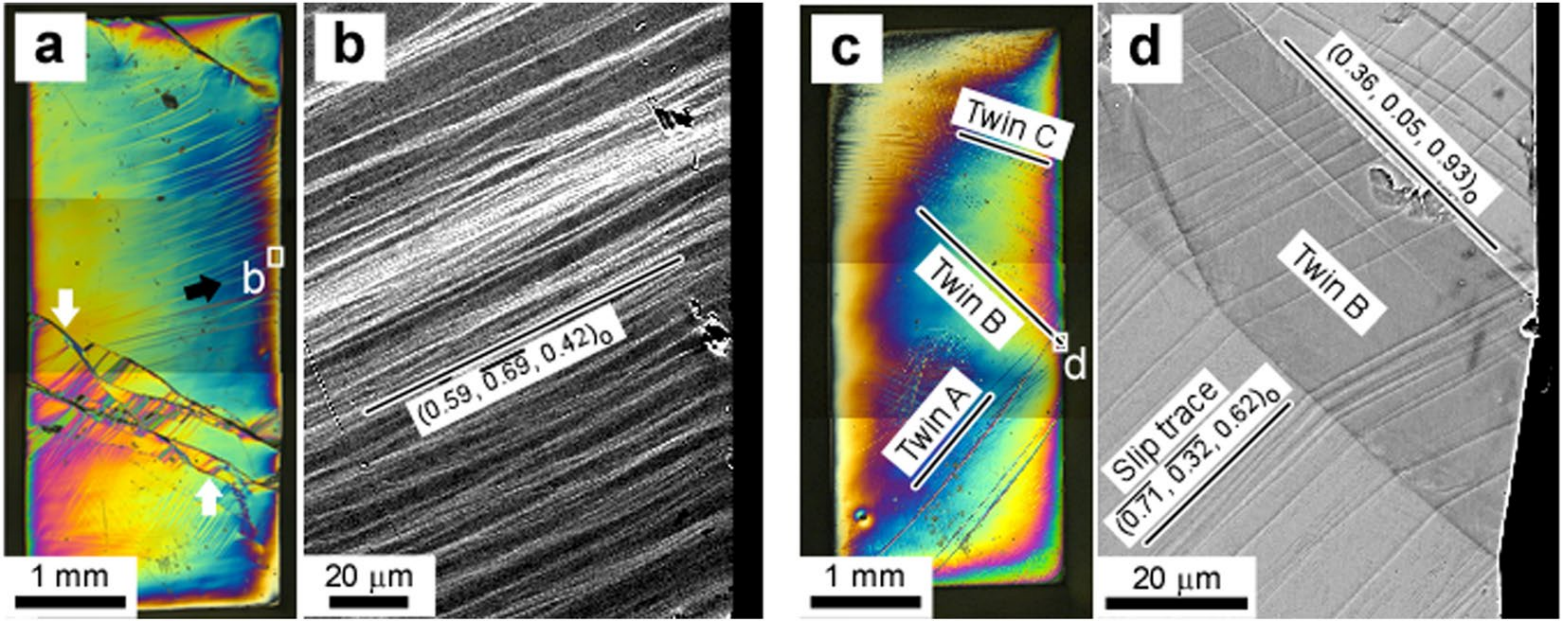
Surface traces in samples that exhibited dislocation slip and deformation twinning. (**a**) OM micrograph and (**b**) SEM-BSE image of Sample #4 after compression, which showed only dislocation slip as plastic deformation mode. Wavy slip traces were observed. The rectangular region in (**a**) corresponds to (**b**). The observed surface plane for (**a**) and (**b**) was ${(0.00,0.95,\overline{0.33})}_{{\rm{o}}}$. (**c**) OM micrograph and (**d**) SEM-BSE image of Sample #1 after compression, which displayed both deformation twinning and dislocation slip. Three types of deformation twins were observed. The rectangular region in (**c**) corresponds to (**d**). The observed surface plane for (**c**) and (**d**) was ${(0.00,0.95,\overline{0.33})}_{{\rm{o}}}$.

**Figure 4:**
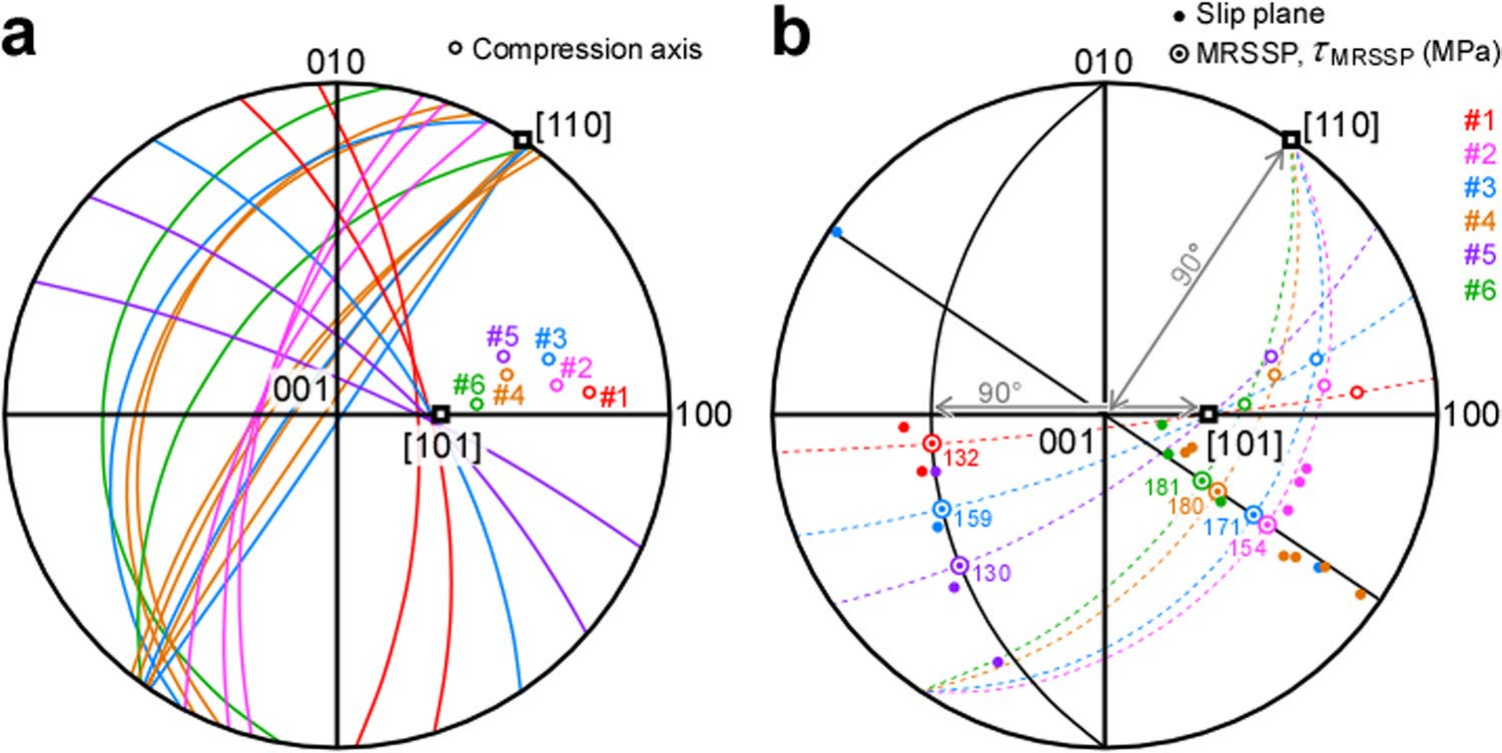
Crystallographic trance analysis of dislocation slip using the stereograms of the α″ martensite centred on 001. (**a**) Observed slip planes are indicated by great circles that pass through either the [110]_o_ or the [101]_o_ zone, which corresponds to the slip direction. (**b**) The maximum resolved shear-stress plane (MRSSP) and the shear yield stress in the MRSSP (*τ*_MRSSP_) along each slip direction are indicated.

In all the samples, wavy trace lines, which correspond to plastic deformation in SMAs, were observed during compression after the second yielding. This means that the wavy trace lines observed by *in-situ* OM were introduced into the single crystalline α″ martensite. Figure 3a shows an OM image (with differential interference contrast (DIC)) of Sample #4 after compression. Several types of curved trace lines, indicated by arrows, were observed. These trace lines sequentially appeared with increasing compression strain, as shown in Supplementary Video 1, and they did not disappear by unloading. The morphology of the trace lines was the same in all the samples. These features suggest that the observed wavy trace lines on the sample surface were due to dislocation slips. The stress required for inducing slip deformation in the single crystalline α″ martensite (*σ*_slip_), i.e. the second yielding stress, of Samples #1–#6 was determined from the stress-strain curves (see Supplementary Fig. S3). The corresponding results are shown in Table 1. Unfortunately, the macroscopic slip planes could not be determined in this study due to the wavy slip lines. However, two-face slip trace analyses were performed on the relatively straight slip lines at the samples edges (see e.g. the slip lines indicated by a black arrow in Fig. 3a), and the local slip planes were accordingly determined. For instance, Fig. 3b shows an image of the edge region of Sample #4 indicated by a white rectangle in Fig. 3a obtained with a scanning electron microscope using backscattered electrons (SEM-BSE). From this image, the slip plane was determined to be ${(0.59,\overline{0.69},0.42)}_{{\rm{o}}}$. The local slip planes introduced by compression in Samples #1–#6 were plotted as great circles in a stereogram of α″ martensite centred on 001 (Fig. 4a). Numerous types of slip plane were observed, which can however be divided into two groups according to whether they lied in the [110]_o_ zone or in the [101]_o_ zone. These two zones correspond to the slip directions since all the observed slip planes were parallel to either [110]_o_ or [101]_o_ directions. Slip planes of the former group were mainly observed in Samples #2, #4 and #6, while those of the latter group were observed in Samples #1 and #5. The great circles that pass through the compression axis and one of the slip directions ([110]_o_ or [101]_o_) are shown with dashed lines in the stereogram in Fig. 4b. The pole that lied on the great circle and at 90° from the slip direction was obtained for all the samples. This pole corresponds to the maximum resolved shear stress plane (MRSSP), which is typically used in the analysis of plastic deformation behaviour of single-crystalline bcc metals and alloys. The operated slip planes observed in this study are shown as a pole indicated with a small closed circle. The operated slip planes for the [110]_o_ slip direction significantly deviated from the MRSSP, whereas those for the [101]_o_ slip direction were close to the MRSSP. The Schmid factor for the MRSSP is shown in Table 1, and the shear yield stress in the MRSSP (*τ*_MRSSP_) in each slip direction was calculated by the *σ*_slip_ and Schmid factor, and the results are given in Fig. 4b. The *τ*_MRSSP_ of the [110]_o_ slip showed the following orientation dependence: ${\tau }_{{\rm{MRSSP}}}^{\#2}$ < ${\tau }_{{\rm{MRSSP}}}^{\#3}$ < ${\tau }_{{\rm{MRSSP}}}^{\#4}$ < ${\tau }_{{\rm{MRSSP}}}^{\#6}$. This means that the *τ*_MRSSP_ increased when the compression axis approached the [001]_o_ direction.

In Sample #1, deformation bands were observed after the second yielding that exhibit features different from those of a dislocation slip: the interface between band and matrix was straight and sharp (Fig. 3c), and the bands were introduced instantaneously (see Supplementary Video 2). All the deformation bands in Sample #1 were introduced simultaneously with an associated sudden stress drop as shown in the stress-strain curve (see Supplementary Fig. S3a). Figure 3d shows an SEM-BSE image of the deformation band in the region corresponding to the rectangle in Fig. 3c. Inside the deformation band, the slip traces appeared before the deformation band was homogeneously sheared. These results strongly suggest that the observed deformation bands in Sample #1 correspond to deformation twinning. As shown in Fig. 3c, three types of deformation twin (Twin A, B and C) were observed, and their twinning plane (*K*_1_) was experimentally determined by a two-face trace analysis as follows: Twin A ${(\overline{0.33},0.94,0.07)}_{{\rm{o}}}$; Twin B (0.36, 0.05, 0.93)_o_; and Twin C ${(0.25,0.97,\overline{0.04})}_{{\rm{o}}}$. The *K*_1_ of Twin A and Twin C were close to {130}_o_, therefore we can consider that they correspond to the previously reported {130}_o_ ${\langle \overline{3}10\rangle }_{{\rm{o}}}$ deformation twinning^41–44^. On the other hand, the *K*_1_ of Twin B was approximately (103)_o_, which has never been reported in orthorhombic crystals^40,58–60^ (e.g. α-uranium, B19 martensite of Ti-Ni base alloys, and α″ martensite of Ti alloys).

**Figure 5:**
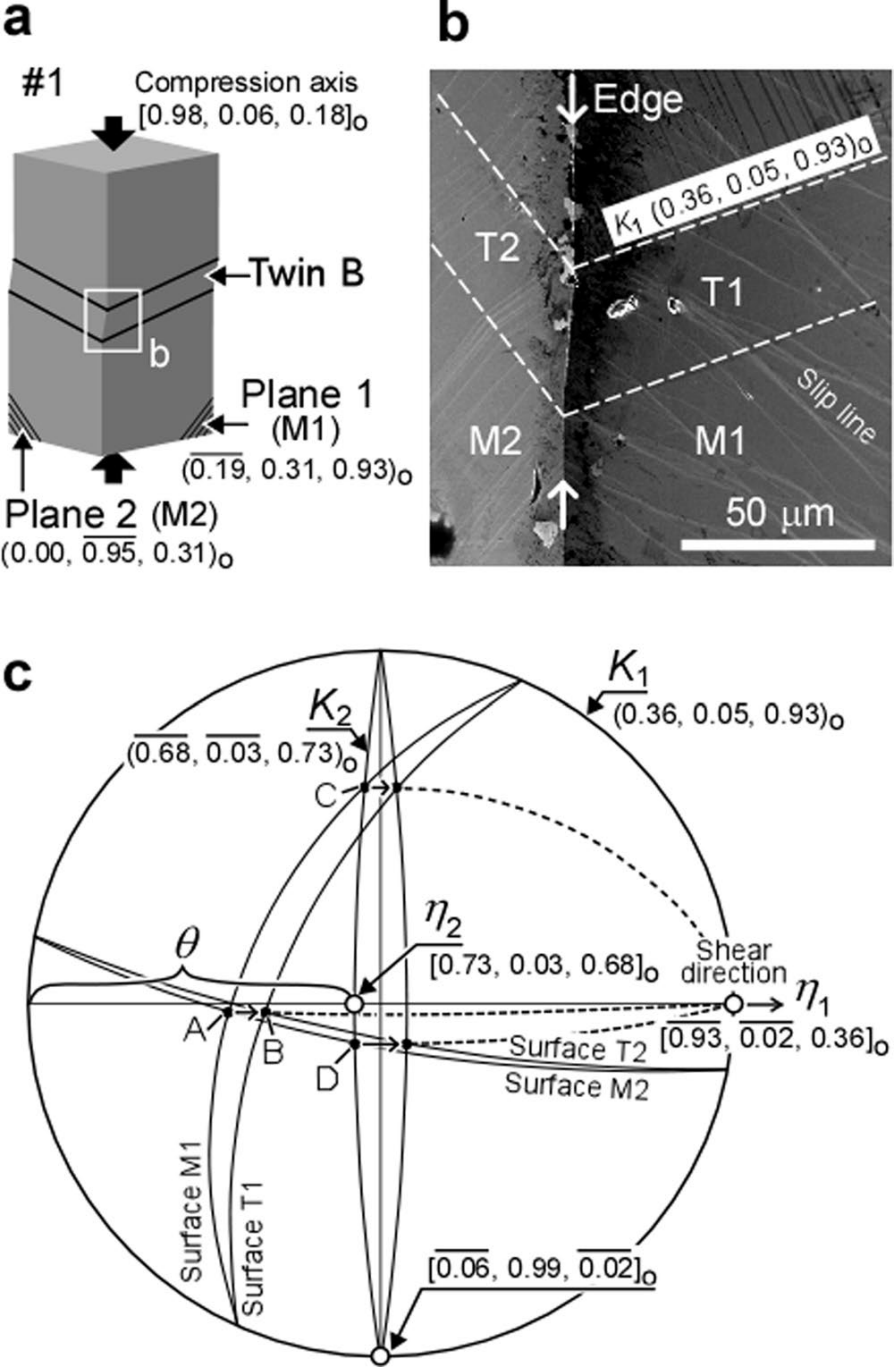
Determination of twinning elements of Twin B in Sample #1 (Fig. 3). (**a**) Crystallographic geometry of Sample #1. (**b**) SEM image of the rectangular region shown in (**a**), corresponding to Twin B. The dashed lines indicate the twinning plane (*K*_1_). T1 and T2 are Surfaces 1 and 2 after distortion by Twin B, respectively. ‘M’ and ‘T’ correspond to matrix and twin, respectively. (**c**) Stereographic projection of Surfaces M1, M2, T1 and T2 onto the *K*_1_. The shear direction (*η*_1_) was determined by Points A and B, while the great circle passing through Points C and D corresponds to the conjugate twinning plane (*K*_2_). The conjugate shear direction (*η*_2_) is the intersection between the *K*_2_ and the equator. The magnitude of shear (*s*) was calculated using the angle *θ* according to the equation *s* = 2cot*θ*.

The other twinning elements, i.e. the shear direction *η*_1_, conjugate twinning plane *K*_2_, conjugate shear direction *η*_2_, and magnitude of shear *s*, of the newly discovered twin (Twin B in Fig. 3c and d) were experimentally determined by a method proposed by Greninger and Troiano^61^. Figure 5a shows a schematic image of Sample #1, while a SEM image of Twin B around the edge between Plane 1 and Plane 2 is shown in Fig. 5b. The surface distortion produced by Twin B was measured, and the traces of matrix (M) and twin (T) for Surfaces 1 and 2 (i.e. M1, M2, T1 and T2) were drawn as great circles in the stereogram shown in Fig. 5c. The plane of projection of this stereogram is the *K*_1_. Point A moved to Point B by twinning shear, and the intersection between the great circle passing through Points A and B and the circumference of the stereogram corresponds to the shear direction *η*_1_. Then, the net was rotated until the *η*_1_ lied along the equator. The *K*_2_ corresponds to the great circle connecting Points C and D. The intersection between the *K*_2_ and the equator corresponds to the *η*_2_, and the *s* was determined using the angle *θ* (*s* = 2cot*θ*). Finally, the twinning elements of Twin B were determined as follows: *K*_1_ = (103)_o_, *η*_1_ = ${[\bar{3}01]}_{{\rm{o}}}$, *K*_2_ = ${(\bar{1}01)}_{{\rm{o}}}$, *η*_2_ = [101]_o_, and *s* = 0.3327.

In Sample #1, the remained α″ martensite after unloading was reverse transformed to parent β phase by heating up to 373 K, and *in-situ* OM observation was performed (see Supplementary Fig. S4). Trace lines of reverse transformed parent β phase were observed during heating, and these traces correspond to those of the habit plane observed during the stress-induced martensitic transformation in Sample #1 (Table 1). Most of deformation features such as slip traces and twinning band were retained after the reverse transformation. From the lattice correspondence between the CV5 of α″ martensite phase and parent β phase (see Supplementary Table S2), *K*_1_ of the reverse transformed deformation twinning was determined to be ${(3\bar{3}\bar{2})}_{{\rm{b}}}$, indicating that the {103}_o_ ${\langle \overline{3}01\rangle }_{{\rm{o}}}$ deformation twinning in α″ phase corresponded to the {332}_b_ ${\langle 11\bar{3}\rangle }_{{\rm{b}}}$ twinning in β phase.

## Discussion

Two slip systems with a slip direction parallel to either [110]_o_ or [101]_o_ were operative in the compression of single-crystalline α″ martensite. If the samples were reversely transformed from α″ martensite phase to the parent β phase, these two slip systems in α″ martensite would correspond to <111>_b_ slips in the parent β phase. Dislocation slips along <111>_b_ are widely observed in bcc metals and alloys including the metastable β-Ti alloys (i.e. the parent β phase)^33–38^. Similarly to the <111>_b_ slip in the parent β phase, the slip lines in the α″ martensite were wavy, and the slip planes were located parallel to the slip directions [110]_o_ and [101]_o_. The crystal structure of α″ martensite was orthorhombic; however, it was close to that of the original parent β phase (bcc) owing to the small lattice deformation strain in this alloy^9^. In addition, the slip traces introduced in the α″ martensite did not disappear by the reverse transformation as shown in Supplementary Fig. S4. Therefore, the similarities between the slip systems of the α″ martensite and parent β phases suggests a dislocation slip mechanism in α″ martensite inherited from that of the parent β phase.

**Figure 6:**
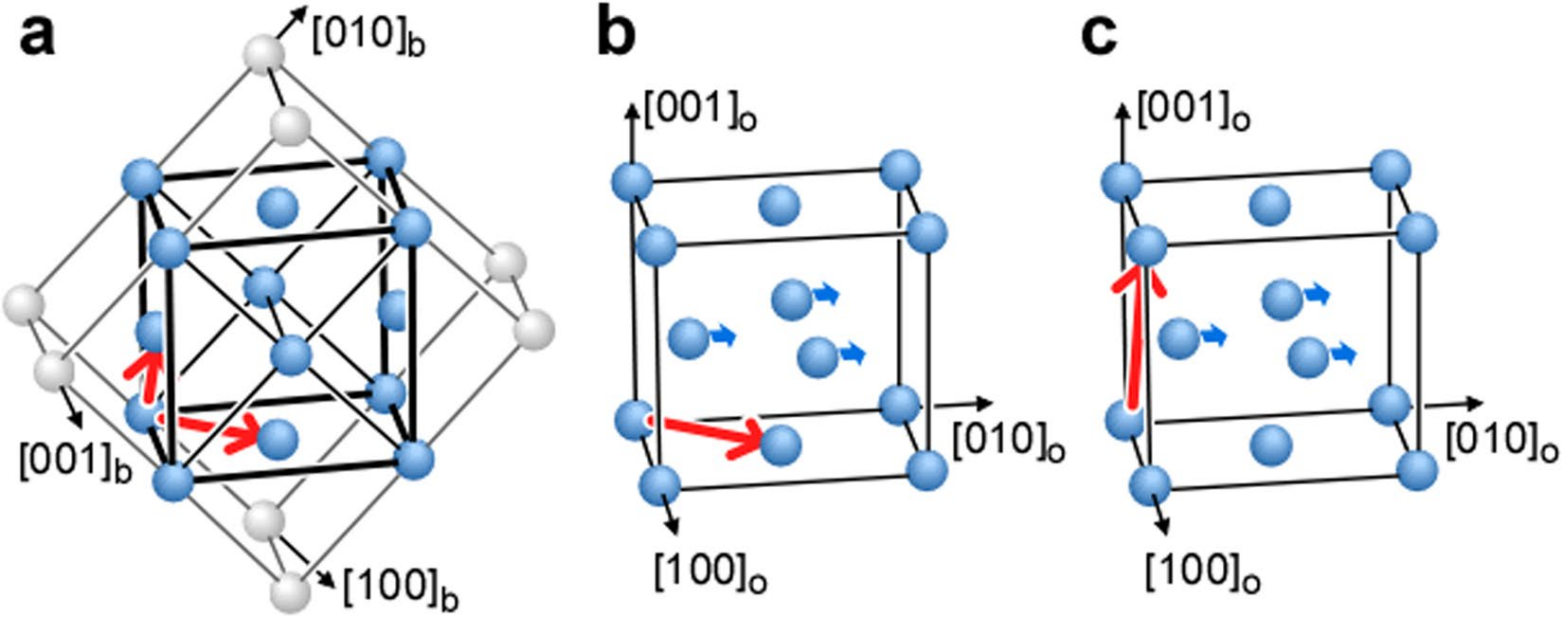
Relationship between the Burgers vector (//slip direction) and basal plane shuffling in α″ martensite. Burgers vectors of the (**a**) <111>_b_ slip (***b*** = 1/2 <111>_b_), (**b**) [110]_o_ slip (***b*** = 1/2 [110]_o_) and (**c**) [101]_o_ slip (***b*** = [101]_o_). The Burgers vector of the [101]_o_ slip is perpendicular to the basal plane shuffling indicated by blue arrows.

While the dislocation slip along [110]_o_ in α″ martensite has been observed in the tensile-deformed Ti-20 mol%Nb alloy^41^, there is no report on dislocation slips along [101]_o_ in α″ martensite. The Burgers vectors (which are parallel to the slip direction) of <111>_b_ slips in the parent β phase, and [110]_o_ and [101]_o_ slips in the α″ martensite phase are indicated in Fig. 6a,b and c, respectively. As shown in Fig. 6c, the Burgers vector of the [101]_o_ slip is ***b*** = [101]_o_, and is thus perpendicular to the shuffling of the (001)_o_ basal plane along the adjacent [010]_o_ direction as a result of the martensitic transformation. In addition, in the case of a perfect dislocation (i.e. with a Burgers vector equal to a translation vector of the lattice), the magnitude of ***b*** for this [101]_o_ slip is about twice as large as that of the [110]_o_ and <111>_b_ slips. We consider that this perfect dislocation can be dissociated into two partial dislocations and a planar fault. However, more work is needed to understand the dislocation dissociation, and systematic studies by transmission electron microscopy (TEM) are under way in our group.

All of the observed deformation twins in this study correspond to the {332}_b_ ${\langle 11\bar{3}\rangle }_{{\rm{b}}}$ twin in the parent β phase. The twinning elements of the observed twins (Twins A, B and C) and {332}_b_ ${\langle 11\bar{3}\rangle }_{{\rm{b}}}$ twin in the parent β phase are given in Supplementary Table S3. Recently, the relationship between the {130}_o_ ${\langle \overline{3}10\rangle }_{{\rm{o}}}$ twinning in the α″ martensite and the {332}_b_ ${\langle 11\bar{3}\rangle }_{{\rm{b}}}$ twinning in the parent β phase has been reported^41–44^. Castany and co-workers^43,44^ insisted that the {332}_b_ ${\langle 11\bar{3}\rangle }_{{\rm{b}}}$ twinning in the plastic deformed β-Ti SMAs is caused by the reverse transformation of the {130}_o_ ${\langle \overline{3}10\rangle }_{{\rm{o}}}$ twinning. This latter twinning corresponds to a plastic deformation as a result of the stress-induced transformation of the parent β phase into the α″ martensite phase during loading, which then underwent a reverse transformation to a {332}_b_ ${\langle 11\bar{3}\rangle }_{{\rm{b}}}$ twinning by unloading. Twins A and C observed in this study belong to the {130}_o_ ${\langle \overline{3}10\rangle }_{{\rm{o}}}$ twinning mode, and thus support the claim by Castany *et al*. On the other hand, although the newly discovered {103}_o_ ${\langle \overline{3}01\rangle }_{{\rm{o}}}$ twinning (Twin B) would, like Twins A and C, correspond to the {332}_b_ ${\langle 11\bar{3}\rangle }_{{\rm{b}}}$ twinning if the sample underwent a reverse transformation into the parent β phase, it is intrinsically different from the {130}_o_ ${\langle \overline{3}10\rangle }_{{\rm{o}}}$ twinning. Tobe *et al*.^41,42^ reported that a complicated ‘structure shuffling’ is necessary for the operation of a {130}_o_ ${\langle \overline{3}10\rangle }_{{\rm{o}}}$ twinning because α″ martensite is a double-lattice structure. An even more complicated structure shuffling is expected in the case of the {103}_o_ ${\langle \overline{3}01\rangle }_{{\rm{o}}}$ twinning since the *η*_1_ of this twin (${\langle \overline{3}01\rangle }_{{\rm{o}}}$) is perpendicular to the α″ martensite basal plane shuffling along <010>_o_. Further observations of the twinning interface by high-resolution TEM are necessary to clarify the {103}_o_ ${\langle \overline{3}01\rangle }_{{\rm{o}}}$ twinning formation mechanism, including the basal plane shuffling.

**Figure 7:**
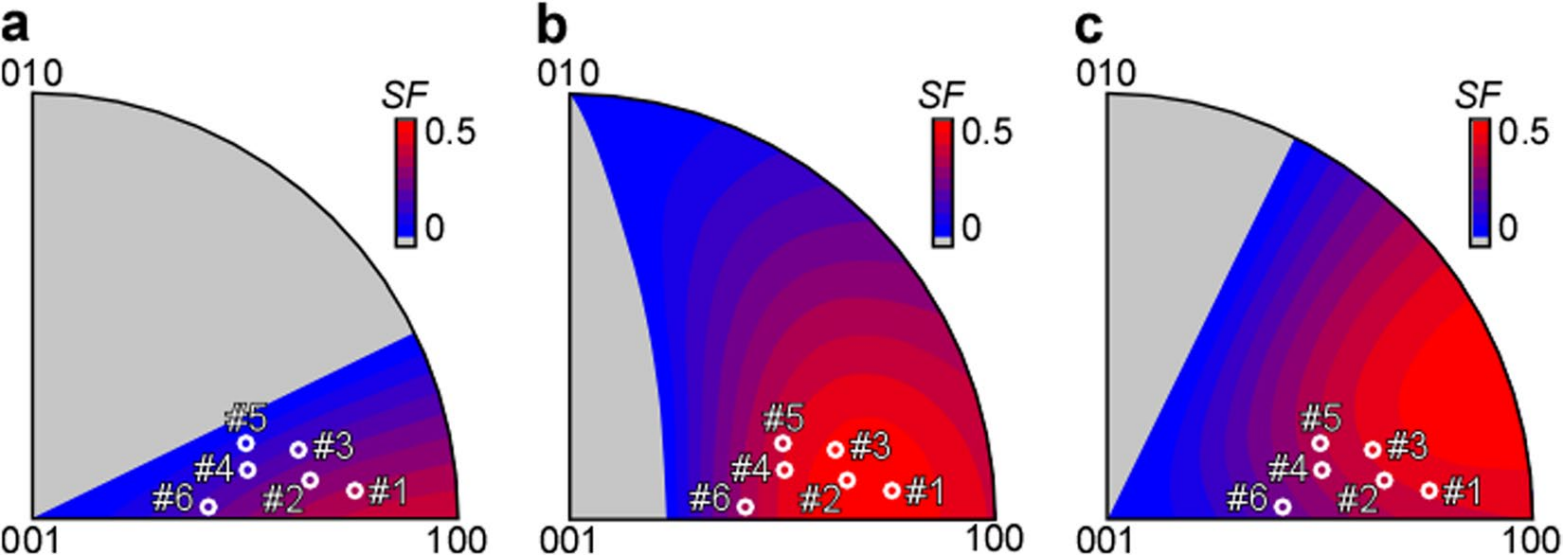
Relationship between the compression axis and the Schmid factor of the observed twinning systems. (**a**) ${(\overline{1}30)}_{{\rm{o}}}$[310]_o_ twinning (Twin A), (**b**) (103)_o_ ${[\overline{3}01]}_{{\rm{o}}}$ twinning (Twin B) and (**c**) (130)_o_ ${[\overline{3}10]}_{{\rm{o}}}$ twinning (Twin C).

Figure 7 shows the value of the Schmid factor for the three observed twinning systems as a function of the compression axis. Sample #1 exhibits the largest Schmid factor for all the operated twinning systems: 0.32 for ${(\overline{1}30)}_{{\rm{o}}}$ [310]_o_ twinning (Twin A), 0.41 for (130)_o_ ${[\overline{3}10]}_{{\rm{o}}}$ twinning (Twin C), and 0.49 for (103)_o_ ${[\overline{3}01]}_{{\rm{o}}}$ twinning (Twin B). This much higher value of the Schmid factor for Sample #1 with respect to the other samples explains why these twinning systems were operative only in Sample #1. In the other samples, two dislocation slip systems ([110]_o_ and [101]_o_ slips) were the predominant plastic deformation modes in this study. As shown in Fig. 4b, the deviation of the operated slip plane from the MRSSP was large in the [110]_o_ slip, whereas it was small in the [101]_o_ slip. This means that the slip plane of [101]_o_ slip easily changed depending on the compression axis, and was controlled only by the resolved shear stress. A certain anisotropy of *τ*_MRSSP_ was found with the [110]_o_ slip since *τ*_MRSSP_ increased when the compression axis approached [001]_o_. As mentioned above, the plastic deformation behaviour of the α″ martensite has many similarities with that of the parent β phase. There are some reports on a similar anisotropy of resolved shear stress in the parent β phase of Ti alloys^36–38^. In general, this anisotropy of resolved shear stress is well known as a twinning/anti-twinning sense asymmetry in bcc metals and alloys. In the case of compression, the resolved shear stress becomes lower when the loading axis is <001>_b_, while it becomes higher when the loading axis is <110>_b_. This feature is successfully explained by a non-planar core structure of screw dislocations in bcc metals and alloys^62^. The origin of the anisotropy of the *τ*_MRSSP_ observed in the α″ martensite is probably closely related to that in the parent β phase since the compression axis of Sample #2 (which exhibited the lowest *τ*_MRSSP_ value of 154 MPa) before loading was close to [001]_b_, and that of Samples #3 (*τ*_MRSSP_ = 171 MPa), #4 (*τ*_MRSSP_ = 180 MPa) and #6 (*τ*_MRSSP_ = 181 MPa) to [110]_b_ (or [111]_b_) as shown in Fig. 2a.

To completely understand the orientation dependence of the plastic deformation in α″ martensite, further experiments with a compression axis along [010]_o_ are necessary. However, only the favourable variant of single-crystalline α″ martensite is obtained by stress-induced martensitic transformation of a single crystal of the parent β phase (i.e. only the CV5 is available in compression). This means that the compression axis in the α″ single crystal always approaches [100]_o_ because the lattice deformation strain by the martensitic transformation along this direction has the largest negative value^9^. Moreover, even if a α″ martensite sample with a compression axis along [010]_o_ was obtained, the compression axis of the sample would be readily converted to [100]_o_ by the reorientation of the martensite variant. The lattice deformation strain along [010]_o_ has the largest positive value, therefore the plastic deformation of the α″ martensite along [010]_o_ is available in tension, not in compression.

Dislocation slip discussed in this study corresponded to the macroscopic slip deformation which was observed as slip traces by OM and SEM. On the other hand, the microscopic slip deformation was possibly introduced to the parent phase and/or stress-induced martensite phase by the movement of habit plane during the stress-induced martensitic transformation. This microscopic slip deformation should play an important role in the stress-induced martensitic transformation, therefore, the further study for the microscopic slip deformation is necessary to obtain the stable superelasticity.

In summary, single-crystalline α″ martensite was successfully obtained by a stress-induced martensitic transformation from a single crystal of the parent β phase, and the plastic deformation behaviour of the different α″ martensite single crystals was systematically investigated. The operative plastic deformation modes in α″ martensite were as follows: (1) dislocation slip along [110]_o_, (2) dislocation slip along [101]_o_, (3) {130}_o_ ${\langle \overline{3}10\rangle }_{{\rm{o}}}$ twinning, and (4) (103)_o_ ${[\overline{3}01]}_{{\rm{o}}}$ twinning. To the best of our knowledge, this is the first study that identified (2) and (4) as plastic deformation mechanisms in α″ martensite. The slip systems (1,2) and deformation twinnings (3,4) correspond to the <111>_b_ slip and the {332}_b_ ${\langle 11\overline{3}\rangle }_{{\rm{b}}}$ twinning in the parent β phase, respectively. The plastic deformation behaviour of the α″ martensite phase is thought to have inherited from that of the parent β phase because of the several similarities observed between the plastic deformation behaviour of both phases. To fully understand the formation process of the newly discovered [101]_o_ slip and (103)_o_ ${[\overline{3}01]}_{{\rm{o}}}$ twinning, the development of new models, including the basal plane shuffling of α″ martensite, is necessary.

## Method

A polycrystalline Ti-27 mol%Nb alloy rod was fabricated by Ar-arc melting using a high purity Ti (99.99%) and Nb (99.9%), and was used as mother alloy. Single crystals were prepared by an optical floating zone method with a grow rate of 5 mm/h under high-purity Ar flow. The single-crystal orientation was determined by X-ray back Laue diffraction. Rectangular specimens (3 × 3 × 6 mm^3^) were cut by a precision diamond wheel cutter for the compression tests. Specimens were solution treated at 1173 K for 3.6 ks in an Ar atmosphere followed by water quenching, and the oxidised surface layer that formed by water quenching was removed by chemical etching at 333 K with a solution of HF:HNO_3_:H_2_O = 7:8:10 in volume. The final specimen size, after etching and before the compression tests, was approximately 2 × 2 × 5 mm^3^. The chemical composition of the samples after the solution treatment was determined by an inductively coupled plasma analysis (Shimadzu, ICPS-8100) and inert gas fusion method (Horiba, EMGA-930). Cyclic loading-unloading compression tests were performed using an Instron-type mechanical testing machine (Shimadzu, Autograph AG-20 kNXPlus) at room temperature with a nominal strain rate of 3.3 × 10^−4^ s^−1^. The sample surface was observed before and after the compression test by an optical microscope (OM) with a differential interference contrast (DIC) mode (Keyence, VHX-100 F). *In-situ* OM observation with DIC mode during heating from room temperature to 373 K was performed for the specimen after compression. *In-situ* video OM observations of two adjacent surfaces parallel to the compression axis were also recorded during the compression tests by two high-resolution charge-coupled device (CCD) camera systems (Shodensha, GR130XGA3). The habit planes were determined by two-face trace analyses. The compression strain was precisely measured from the videos using a motion analyser software (Keyence, VW-H2MA). The deformation microstructure was observed by a scanning electron microscope (SEM) equipped with a back-scattered electron (BSE) detector (Hitachi, SU5000). The following lattice parameters of the parent β (*a*_b_) and α″ martensite (*a*_o_, *b*_o_ and *c*_o_) phases reported in a previous study^9^ were used for the calculation of the interaction energy (*U*), lattice deformation strain due to the stress-induced martensitic transformation, habit plane using the phenomenological theory of martensite crystallography (PTMC), and coordinate conversion between β and α″ phases: *a*_b_ = 0.3290 nm, *a*_o_ = 0.3225 nm, *b*_o_ = 0.4770 nm and *c*_o_ = 0.4615 nm.

## Electronic supplementary material

Supplementary Information

Supplementary_Video_1

Supplementary_Video_2
