# Supplementary material for: Plastic deformation behaviour of single-crystalline martensite of Ti-Nb shape memory alloy
Source: Sci Rep. 2017 Nov 16;7:15715. doi: 10.1038/s41598-017-15877-6 (PMC5691214; doi:10.1038/s41598-017-15877-6)
Supplement: Supplementary file 1 — Supplementary Information [file 41598_2017_15877_MOESM1_ESM.pdf]

Supplementary Information

Plastic deformation behaviour of single-crystalline martensite of Ti-Nb shape memory alloy

Masaki Tahara [1, 2 *], Nao Okano [3, a], Tomonari Inamura [1, 2], and Hideki Hosoda [1, 2]

[1] Laboratory for Materials and Structures, Institute of Innovative Research, Tokyo Institute of Technology, 4259 Nagatsutacho, Midori-ku, Yokohama 226-8503, Japan.

[2] Laboratory for Future Interdisciplinary Research of Science and Technology, Institute of Innovative Research, Tokyo Institute of Technology, 4259 Nagatsutacho, Midori-ku, Yokohama 226-8503, Japan.

[3] Graduate Student, Tokyo Institute of Technology, 4259 Nagatsutacho, Midori-ku, Yokohama 226-8503, Japan.

[*] Corresponding author: Laboratory for Materials and Structures, Institute of Innovative Research, Tokyo Institute of Technology, 4259 Nagatsutacho, Midori-ku, Yokohama 226-8503, Japan.
(E-mail: tahara.m.aa@m.titech.ac.jp)

[a] Current affiliation: Nippon Steel & Sumitomo Metal Corporation, 1-8 Fusoucho, Amagasaki, Hyogo 660-0891, Japan.

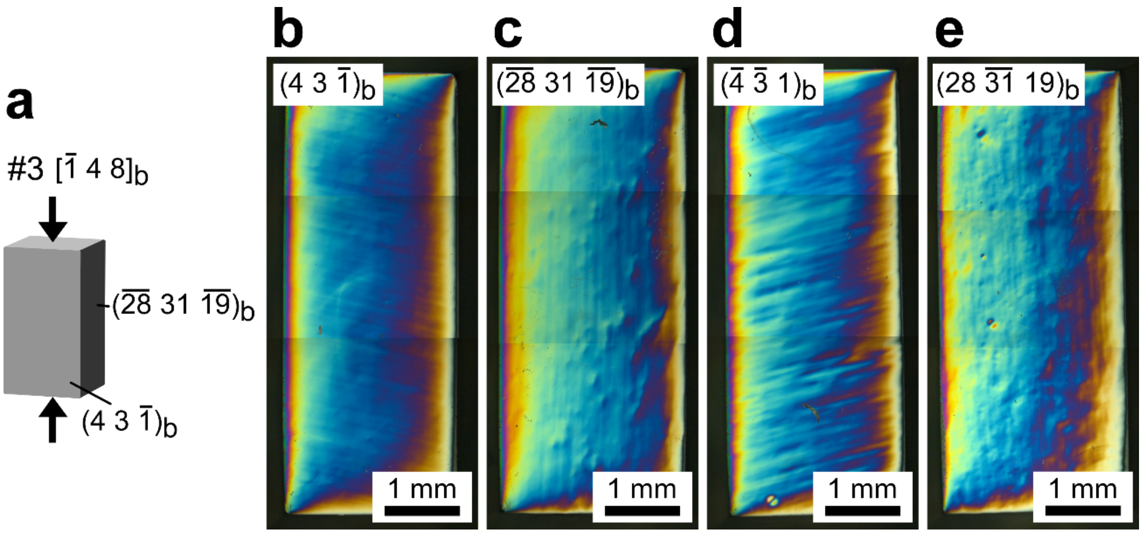

Figure S1. OM micrographs of Sample #3 before compression. (**a**) Crystallographic geometry of Sample #3. (**b** – **e**) Optical micrographs of Sample #3 taken in a differential interference contrast (DIC) mode. The entire sample exhibited a smooth surface before compression, corresponding to the parent β phase.

Table S1. Chemical composition of the single-crystalline sample after the solution treatment (mol%).

	Ti	Nb	Fe	O	N
Average	Bal.	27.265	0.011	0.209	0.013
#1	Bal.	26.398	0.011	0.227	0.010
#2	Bal.	27.717	0.011	0.250	0.017
#3	Bal.	28.333	0.011	0.200	0.017
#4	Bal.	27.590	0.011	0.199	0.011
#5	Bal.	26.112	0.011	0.167	0.012
#6	Bal.	27.438	0.011	0.209	0.013

Table S2. Lattice correspondence between the parent β and α″ martensite phases, and the habit planes of each martensite variant. 'CV' means the lattice correspondence variant.

CVi	$[100]_o$	$[010]_o$	$[001]_o$	Habit plane (+)	Habit plane (−)
CV1	$[100]_b$	$[011]_b$	$[0\bar{1}1]_b$	$(0.7305, 0.4829, 0.4829)_b$	$(0.7287, \overline{0.4842}, \overline{0.4842})_b$
CV2	$[\bar{1}00]_b$	$[0\bar{1}1]_b$	$[011]_b$	$(0.7296, \overline{0.4848}, 0.4823)_b$	$(0.7296, 0.4823, \overline{0.4848})_b$
CV3	$[010]_b$	$[101]_b$	$[10\bar{1}]_b$	$(0.4823, 0.7296, 0.4848)_b$	$(\overline{0.4848}, 0.7296, \overline{0.4823})_b$
CV4	$[0\bar{1}0]_b$	$[10\bar{1}]_b$	$[101]_b$	$(0.4829, 0.7304, \overline{0.4829})_b$	$(\overline{0.4842}, 0.7287, 0.4842)_b$
CV5	$[001]_b$	$[110]_b$	$[\bar{1}10]_b$	$(0.4829, 0.4829, 0.7305)_b$	$(\overline{0.4842}, \overline{0.4842}, 0.7287)_b$
CV6	$[00\bar{1}]_b$	$[\bar{1}10]_b$	$[110]_b$	$(\overline{0.4848}, 0.4823, 0.7296)_b$	$(0.4823, \overline{0.4848}, 0.7296)_b$

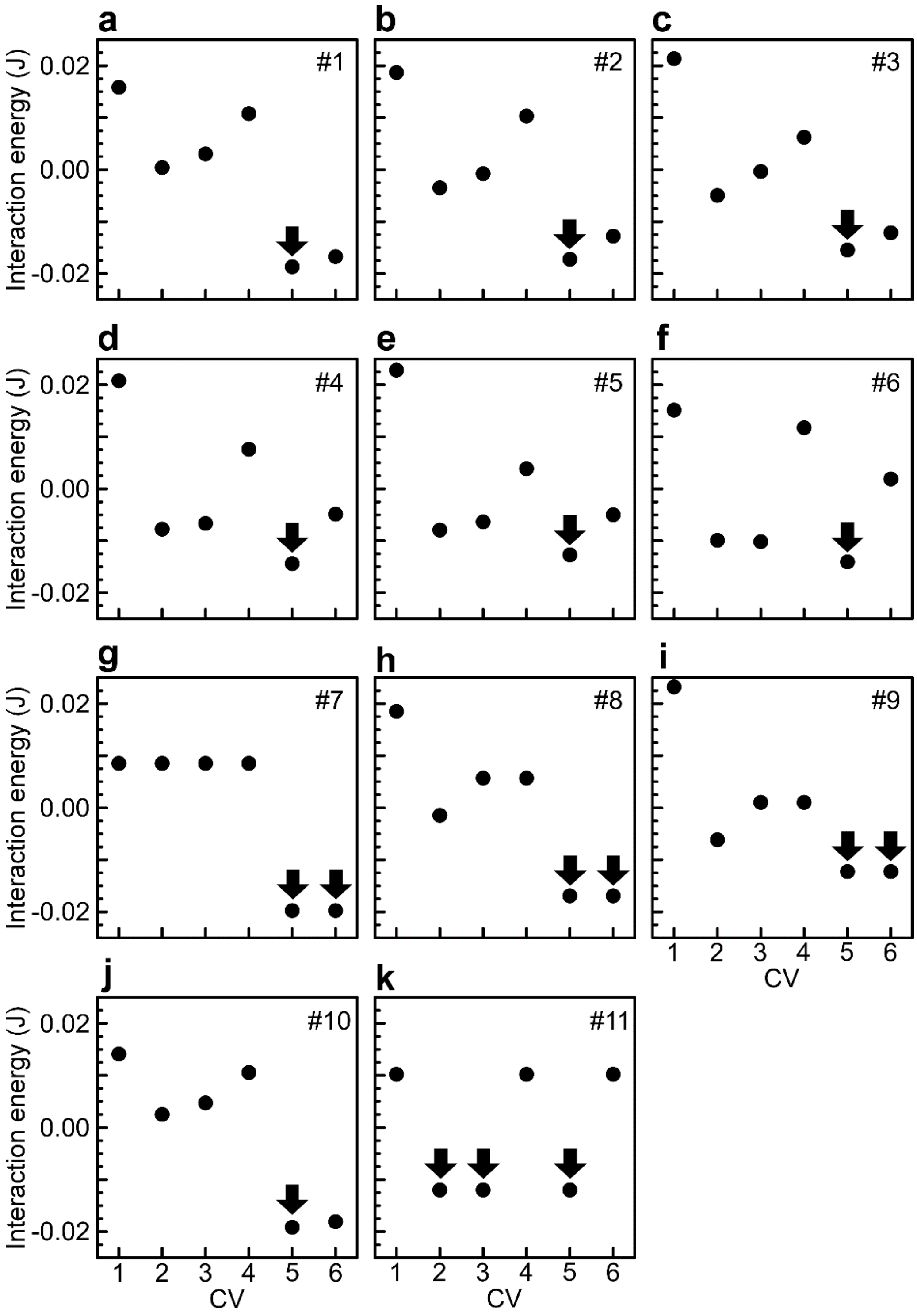

Figure S2. Calculated interaction energy (*U*) between the uniaxial compression stress and the lattice deformation strain along the compression axis for the eleven samples (#1 – #11). The CV(s) which has(have) the largest negative value of *U* is(are) the favourable variant(s), and are indicated by arrows.

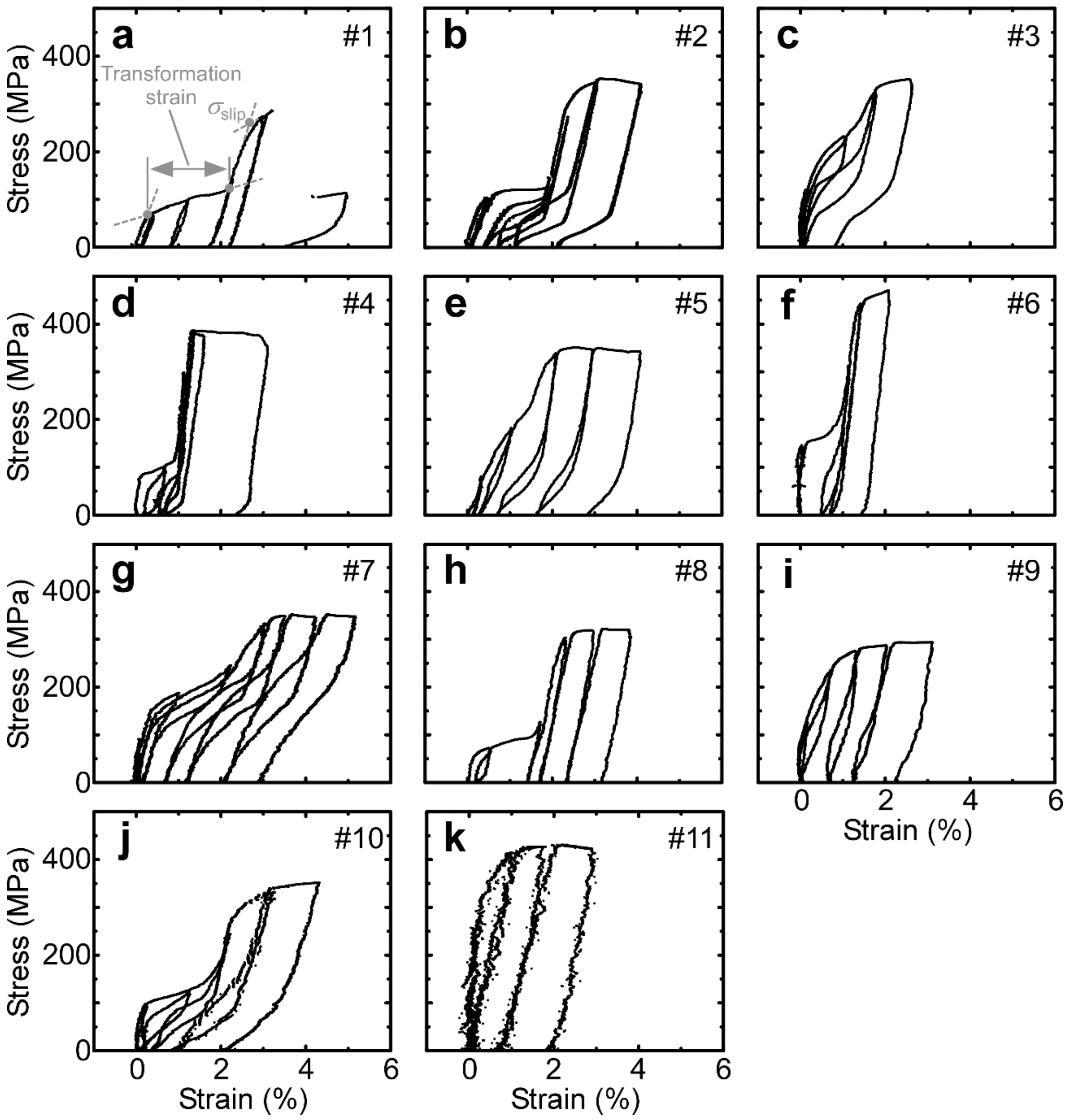

Figure S3. Stress-strain curves obtained by cyclic loading-unloading compression tests. The transformation strain and second yield stress (σ_{slip}) were determined by using the tangent lines as shown in (**a**).

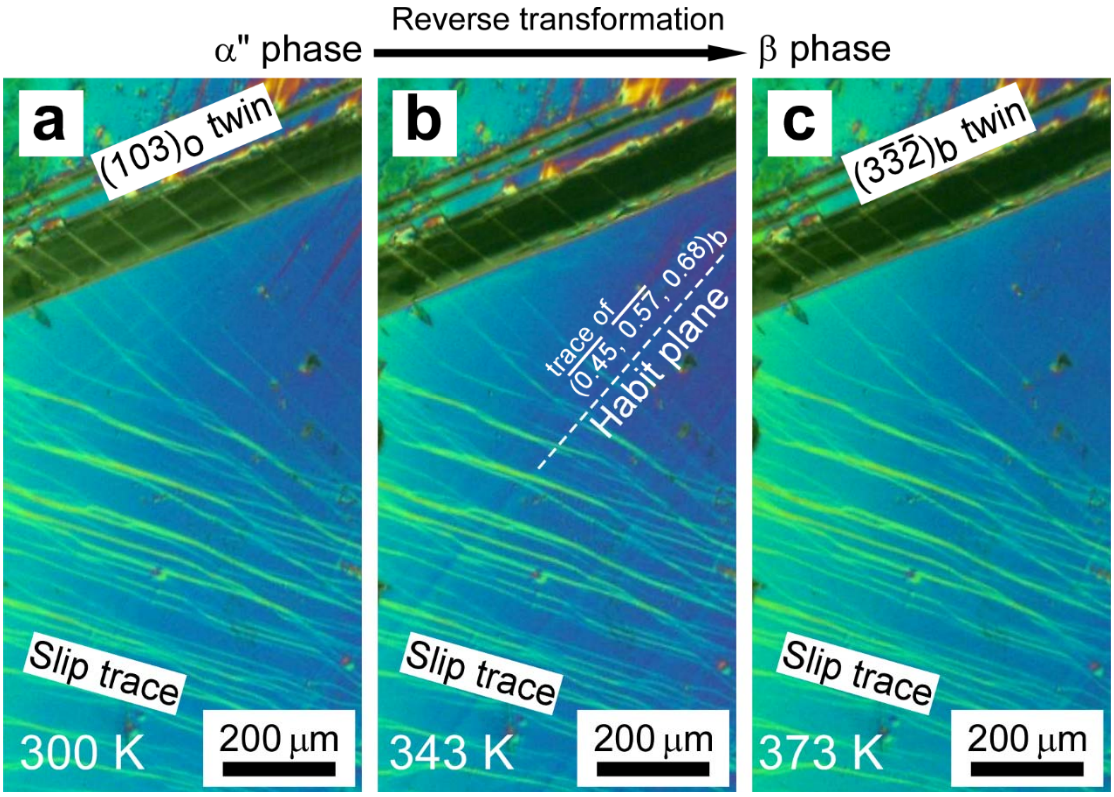

Figure S4. In situ OM observation during heating from 300 K to 373 K for Sample #1 after compression test. Optical micrographs of Sample #1 after compression taken in a differential interference contrast (DIC) mode at (a) 300 K, (b) 343 K, and (c) 373 K. Observed surface plane was $(0.00, \overline{0.95}, 0.31)_o$ and $(8\ \overline{16}\ 5)_b$ for α″ martensite phase and β parent phase, respectively.

Table S3. Twinning elements (K_1, K_2, η_1, η_2 and s) of the operative twinning systems in Sample #1. The twinning elements of the $\{332\}_b\langle 11\bar{3}\rangle_b$ twin in the parent β phase are also shown for reference.

Twinning mode	Observed twinning in Sample #1	K_1	K_2	η_1	η_2	s
$(\bar{1}30)_o[310]_o$	Twin A	$(\bar{1}30)_o$	$(110)_o$	$[310]_o$	$[\bar{1}10]_o$	0.2746
$(103)_o[\bar{3}01]_o$	Twin B	$(103)_o$	$(\bar{1}01)_o$	$[\bar{3}01]_o$	$[101]_o$	0.3327
$(130)_o[\bar{3}10]_o$	Twin C	$(130)_o$	$(\bar{1}10)_o$	$[\bar{3}10]_o$	$[110]_o$	0.2746
$\{332\}_b\langle 11\bar{3}\rangle_b$	—	$\{332\}_b$	$\{11\bar{2}\}_b$	$\langle 11\bar{3}\rangle_b$	$\langle 111\rangle_b$	0.3536

Supplementary Video

Supplementary Video1. Slip deformation observed in Sample #4. The observed plane is $(0.00, \overline{0.95}, 0.31)_o$, and the movie starts after the second yielding. The wavy slip trace propagated from the upper side.

Supplementary Video2. Deformation twinning observed in Sample #1. The observed plane is $(0.00, \overline{0.95}, 0.31)_o$, and the movie starts after the second yielding. The deformation twinnings with straight and parallel surface reliefs were formed instantaneously.
